# GENERATIONAL TRANSMISSION OF SOCIAL RELATIONS: FINDINGS FROM MULTIPLE US LONGITUDINAL STUDIES

**DOI:** 10.1093/geroni/igac059.148

**Published:** 2022-12-20

**Authors:** Rita Hu, Toni Antonucci, Shevaun Neupert

## Abstract

This symposium provides diverse findings documenting the long reach of social relations over generations. Ali and Rohner examine data from 41 adult offspring showing that recalled perception of rejection of parents during childhood are associated with fewer positive caregiving behaviors and social interactions with their now aging parents. Using three waves of longitudinal data over 23 years, Manalel, Cleary & Antonucci examine changes in composition, proximity, and contact frequency in social relations among 193 participants who were 8-12 years old at wave 1 (1992). Findings indicate increased diversity from wave 1 to 2 and increased stability from Wave 2 to 3, reflecting normative life transitions. Gender and race differences were also evident. Suitor, Gilligan, Frase & Stepniak examine 725 adult (aged 30-60) children’s experience of their mother’s advice concerning experienced depression and whether these differ by race, age, and gender. While there were no age differences, men, regardless of race and black daughters receiving advice had higher levels of depression but this had little effect white daughters. Finally, Hu and Antonucci use the Social Relations Study to examine the longitudinal association between social ties and self-esteem. They examined 553 people who were 13-77 at Wave 1 in 1992. Findings indicate that network closeness matters with increases in weak and close, but not closest network size related to increase in self-esteem 23 years later. In sum, this symposium offers multiple and diverse perspectives of generations in social relations and their association with well-being over the life span.

